# COVID-19: what has been learned and to be learned about the novel coronavirus disease

**DOI:** 10.7150/ijbs.45134

**Published:** 2020-03-15

**Authors:** Ye Yi, Philip N.P. Lagniton, Sen Ye, Enqin Li, Ren-He Xu

**Affiliations:** Institute of Translational Medicine, and Centre of Reproduction, Development and Aging, Faculty of Health Sciences, University of Macau, Taipa, Macau, China.

**Keywords:** Coronavirus, pneumonia, outbreak, SARS-CoV-2, COVID-19

## Abstract

The outbreak of Coronavirus disease 2019 (COVID-19), caused by severe acute respiratory syndrome (SARS) coronavirus 2 (SARS-CoV-2), has thus far killed over 3,000 people and infected over 80,000 in China and elsewhere in the world, resulting in catastrophe for humans. Similar to its homologous virus, SARS-CoV, which caused SARS in thousands of people in 2003, SARS-CoV-2 might also be transmitted from the bats and causes similar symptoms through a similar mechanism. However, COVID-19 has lower severity and mortality than SARS but is much more transmissive and affects more elderly individuals than youth and more men than women. In response to the rapidly increasing number of publications on the emerging disease, this article attempts to provide a timely and comprehensive review of the swiftly developing research subject. We will cover the basics about the epidemiology, etiology, virology, diagnosis, treatment, prognosis, and prevention of the disease. Although many questions still require answers, we hope that this review helps in the understanding and eradication of the threatening disease.

## Introduction

The Spring Festival on January 25, 2020 has become an unprecedented and unforgettable memory to all Chinese who were urged to stay indoors for all the holiday and for many weeks after due to the outbreak of a novel viral disease. The virus is highly homologous to the coronavirus (CoV) that caused an outbreak of severe acute respiratory syndrome (SARS) in 2003; thus, it was named SARS-CoV-2 by the World Health Organization (WHO) on February 11, 2020, and the associated disease was named CoV Disease-19 (COVID-19) 1. The epidemic started in Wuhan, China, and quickly spread throughout the entire country and to near 50 others all over the world. As of March 2, 2020, the virus has resulted in over 80,000 confirmed cases of COVID-19, with more than 40,000 patients discharged and over 3,000 patients who died. WHO warns that COVID-19 is “public enemy number 1” and potentially more powerful than terrorism 2.

According to PubMed (https://www.ncbi.nlm.nih.gov/pubmed/), in less than two months, over 200 papers have been published on COVID-19 including its virology, epidemiology, etiology, diagnosis, and treatment since the first report on January 7, 2020 that determined the sequence of the virus isolated from multiple patients 3. This review attempts to summarize the research progress in the new and swiftly developing subject area. Whenever possible, we will try to compare COVID-19 with SARS and another CoV-caused disease, Middle East respiratory syndrome (MERS, an outbreak in 2012). We will also discuss what we have learned so far regarding the prevention and prognosis of the disease as well as some remaining yet urgent questions.

## The outbreak

CoVs have been traditionally considered nonlethal pathogens to humans, mainly causing approximately 15% of common colds 4. However, in this century, we have encountered highly pathogenic human CoVs twice, *i.e.*, SARS-CoV and MERS-CoV, which caused an outbreak originally in China in 2003 and Saudi Arabia in 2012, respectively, and soon spread to many other countries with horrible morbidity and mortality 5. Therefore, the current COVID-19 is the third CoV outbreak in the recorded history of humans.

**Figure 1:**
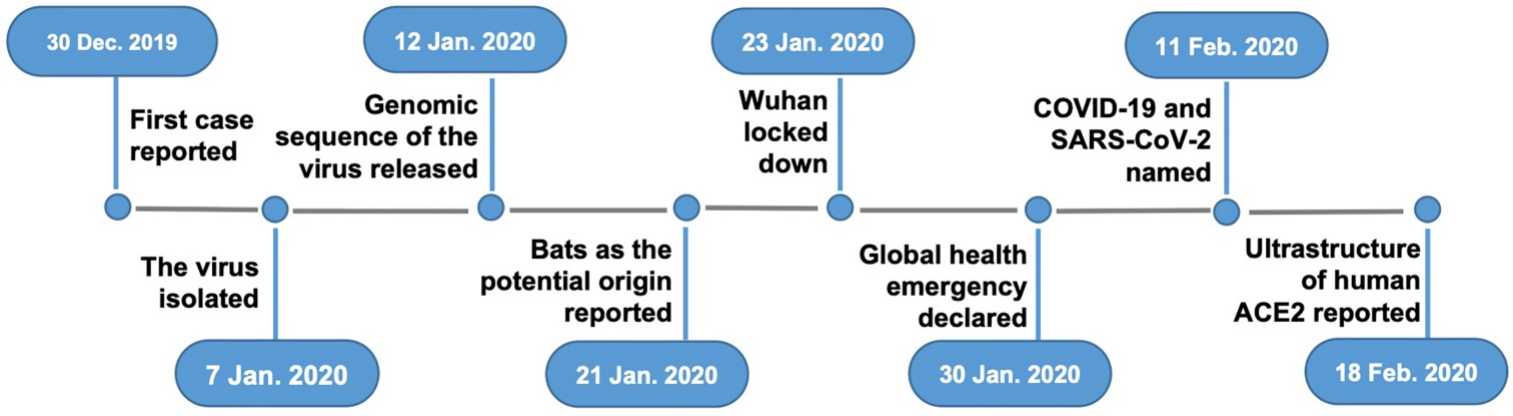
Major events that occurred thus far during the outbreak of COVID-19.

**Figure 2:**
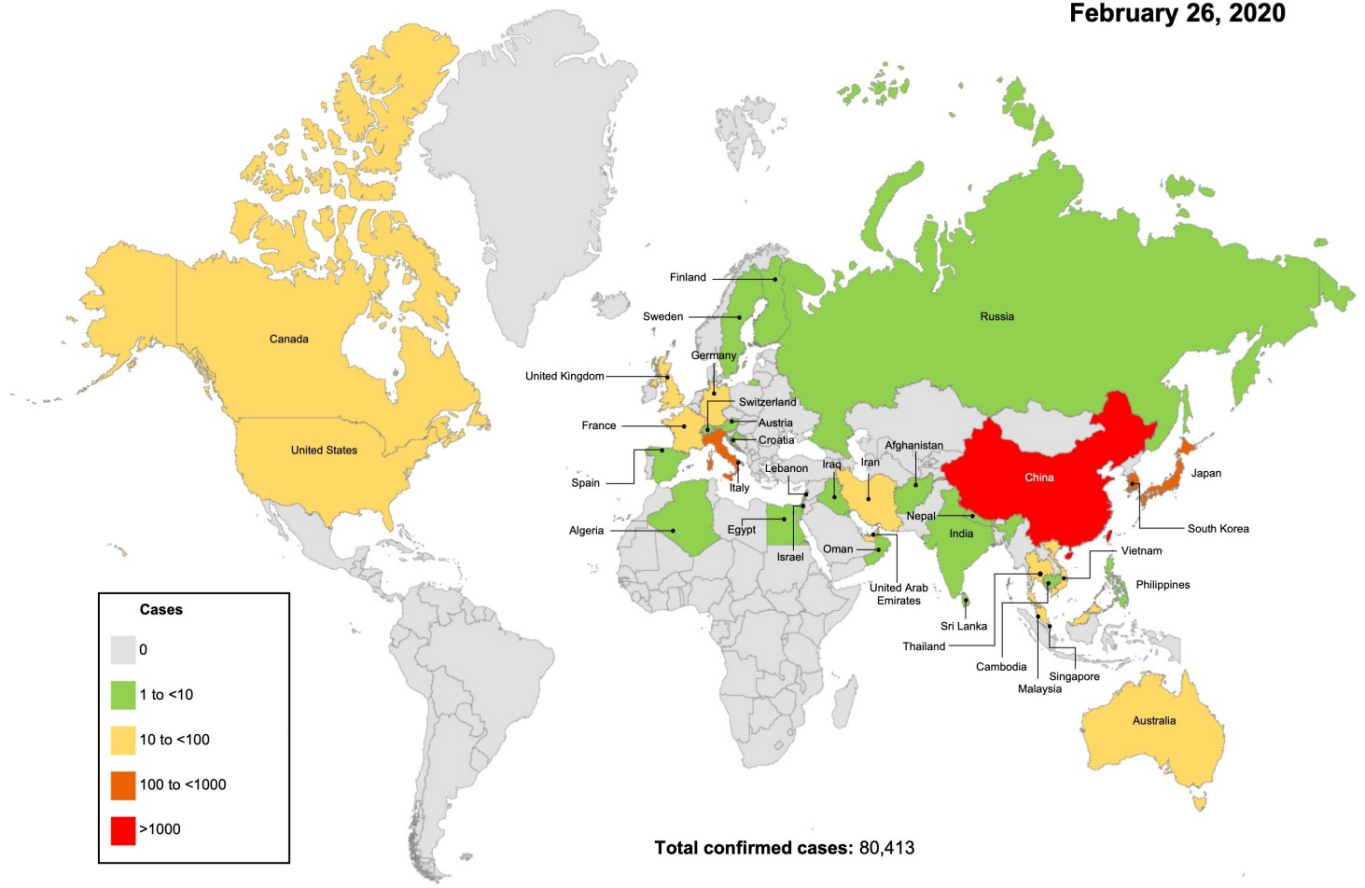
Worldwide distribution of COVID-19 cases on 26 Feb. 2020, according to a coronavirus monitoring system of Johns Hopkins University 9,10.

As shown in Fig. 1, clusters of pneumonia that had unknown origins were first reported from Wuhan on December 31, 2019 to the China National Health Commission 6. Seven days later the sequence of the CoV was released 7. On January 15, 2020 the first fatal case from Wuhan was reported 6. Meanwhile, the epidemic rapidly spread to the neighboring cities, provinces, and countries. On January 20, the infection of health-care providers was reported, suggesting that human-to-human transmission was possible 8. On January 23, the city of Wuhan was locked down with all its public transportation stopped. On January 24 the first clinical study on the disease reported that, out of 41 patients with confirmed cases, only 21 had direct contact with the Wuhan seafood market that was considered the starting site of the infection from an unknown animal source 6. On January 30, WHO declared the outbreak a global health emergency. By the time of this report, the disease has already spread throughout China and near 50 other countries all over the world (Fig. 2). As the situation is rapidly evolving, the final scope and severity of the outbreak remain to be determined.

On February 11, 2020, a multi-center study on 8,866 patients including 4,021 confirmed COVID-19 patients presented a more updated illustration of the epidemic as follows (https://mp.weixin.qq.com/s/UlBi-HX_rHPXa1qHA2bhdA).

- SARS-CoV-2 infected people of all ages, but mainly at the age of 30-65. Almost half (47.7%) of the infected individuals were over 50 years old, very few were under 20, and only 14 infected individuals were under the age of 10.
- SARS-CoV-2 infected more men (0.31/100,000) than women (0.27/100,000).
- COVID-19 expanded in clusters mainly in and around Hubei.
- COVID-19 took an average of 5 (2-9) days from onset to diagnosis. The average incubation period was 4.8 (3.0-7.2) days. The average time from onset to death was 9.5 (4.8-13) days.
- The basic reproductive number (R0) was 3.77 (95% CI: 3.51-4.05), and the adjusted R0 was 2.23-4.82.
- The number of infected people increased exponentially before 23 Jan. 2020, matching the time of massive transportation before the Spring Festival in China.
- The mortality of patients with confirmed cases was 1.44% (95% CI: 1.10-1.86%), and the adjusted mortality of all the patients was 3.06% (95% CI: 2.02-4.59%).
- Three major risk factors for COVID-19 were sex (male), age (≥60), and severe pneumonia.

## SARS-CoV-2

### Etiology

CoVs are a subfamily of large and enveloped viruses containing a single strand of sense RNA. They can be divided into four genera, *i.e*., alpha, beta, gamma, and delta, of which alpha- and beta-CoVs are known to infect humans 11. The envelope spike (S) glycoprotein binds to its cellular receptors angiotensin-converting enzyme 2 (ACE2) and dipeptidyl peptidase 4 (DPP4) for SARS-CoV and MERS-CoV, respectively, and then membrane fusion occurs 12. The viral RNA genome is released into the cytoplasm; after replication of the viral genome, genomic RNA accompanied by envelope glycoproteins and nucleocapsid proteins forms virion-containing vesicles, which then fuse with the plasma membrane to release the virus 13.

The first genomic sequence of SARS-CoV-2 was reported on January 10, 2020 3. SARS-CoV-2 was found to be a new type of beta-CoV with more than 99.98% genetic identity among 10 sequenced samples collected from the original site of the outbreak, the Huanan Seafood Market in Wuhan. SARS-CoV-2 is genetically more similar to SARS-CoV than to MERS-CoV 14-16. Through transmission electron microscopy, SARS-CoV-2 particles were found in ultrathin sections of human airway epithelium 17. Human ACE2 was found to be a receptor for SARS-CoV-2 as well as SARS-CoV 16,18,19. However, the S protein of SARS-CoV-2 binds to human ACE2 more weakly than that of SARS-CoV, which is coincident with the fact that SARS-CoV-2 causes less severe infection in patients than SARS-CoV 14.

SARS-CoV-2 can also form a novel short protein encoded by *orf3b* and a secreted protein encoded by *orf8*. The orf3b of SARS-CoV-2 may play a role in the viral pathogenicity and inhibit the expression of *IFNβ*; however, orf8 does not contain any known functional domain or motif 20. On February 18, 2020, Zhou, *et al.*, reported the cryo-EM structure of the full-length human ACE2 at 2.9 Å resolution in complex with the amino acid transporter B^0^AT1 21. They found that the complex, which had open and closed conformations, was assembled as a dimer and the ACE2-B^0^AT1 complex can bind two S proteins, which provides evidence for CoV recognition and infection. B^0^AT1 may become a therapeutic target for drug screening to suppress SARS-CoV-2 infection.

### The origin and intermediate host

**Figure 3:**
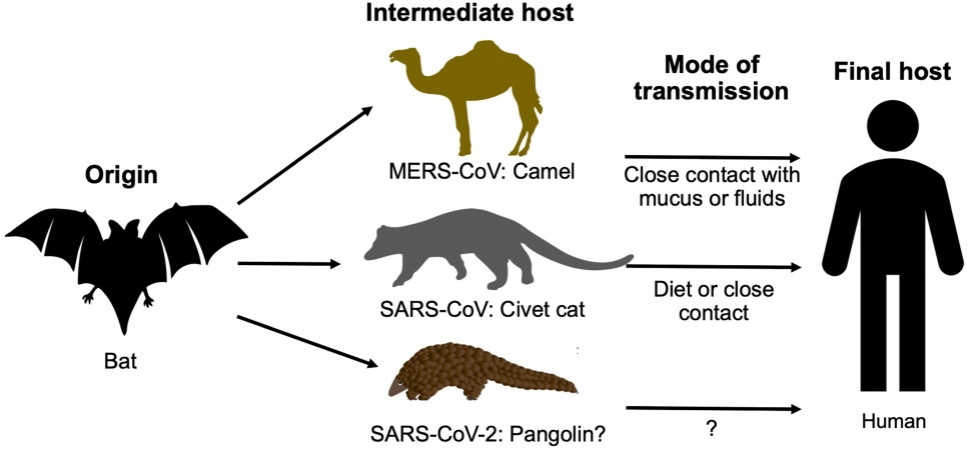
The origins and intermediate hosts of SARS-CoV-2, SARS-CoV, and MERS-CoV.

It has been known that both SARS-CoV and MERS-CoV originated from bats and were transmitted to humans via civet cats and camels, respectively. Through a phylogenetic comparison of SARS-CoV-2 with other CoVs, bats were considered the native host of SARS-CoV-2 as the new virus is 96% identical to two SARS-like CoVs from bats called bat-SL-CoVZX45 and bat-SL-CoVZX21 14-16,19. However, what intermediate host helped the virus cross the species barrier to infect humans remains unknown, and the transmission route is yet to be elucidated. Ji, *et al*., proposed snakes as a carrier of the virus from bats to humans which involved homologous recombination within the S protein 22. According to a study, researchers in Guangzhou, China, suggested that pangolins - long-snouted, ant-eating mammals often used in traditional Chinese medicine - are the potential intermediate host of SARS-CoV-2 based on 99% genetic homology in a CoV discovered in pangolins and SARS-CoV-2 23. However, 1% difference spread all over two genomes is still a big difference; thus, conclusive results for concrete evidence are awaited (Fig. 3).

### Physicochemical properties

The physicochemical properties of SARS-CoV-2 are largely not yet known. SARS-CoV and MERS-CoV can survive *in vitro* for 48 hours in a dry environment and up to 5 days under 20 °C and 40%-50% humidity 24-26. SARS-CoV-2 may possess similar properties. It has been reported that SARS-CoV-2 is sensitive to ultraviolet rays and heat at 56 °C for 30 minutes; ether, 75% ethanol, chlorine-containing disinfectant, peracetic acid, chloroform, and other fatty solvents, but not chlorhexidine, can effectively inactivate the virus 27.

### Immune responses to CoVs

**Figure 4:**
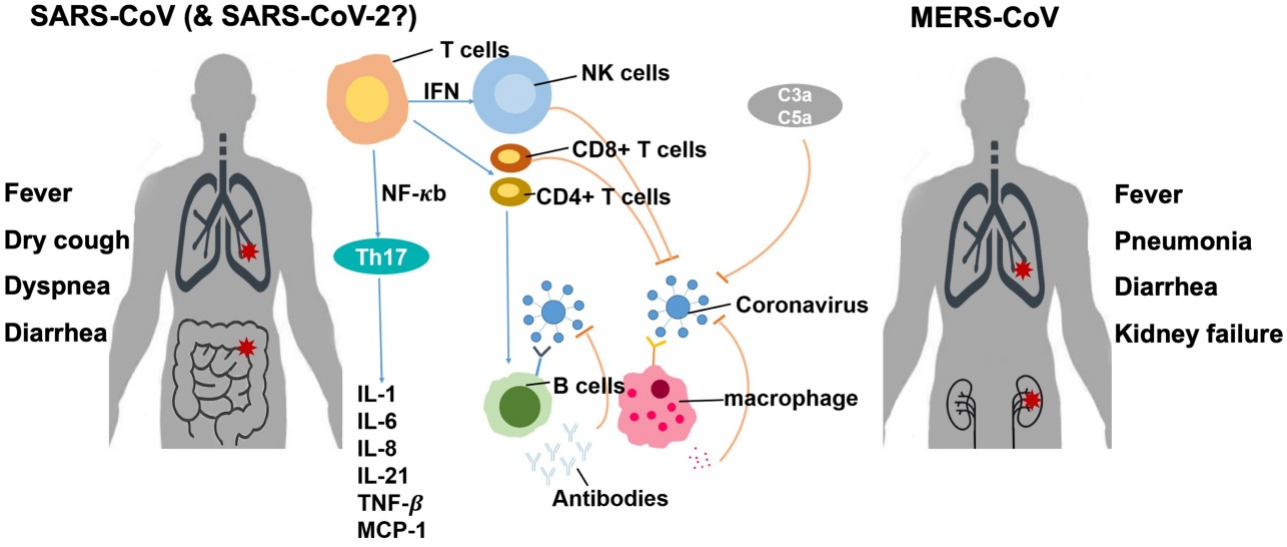
Immune response of the host to coronavirus infection 4, 32-34.

The entire human population generally lacks immunity to SARS-CoV-2 and hence is susceptible to the novel virus. Currently, no detailed study has been reported regarding the immunological response to SARS-CoV-2. Thus, we can only refer to previous studies on other CoVs, especially SARS-CoV and MERS-CoV (Fig. 4). In general, after a virus invades the host, it is first recognized by the host innate immune system through pattern recognition receptors (PRRs) including C-type lectin-like receptors, Toll-like receptor (TLR), NOD-like receptor (NLR), and RIG-I-like receptor (RLR) 28. Through different pathways, the virus induces the expression of inflammatory factors, maturation of dendritic cells, and synthesis of type I interferons (IFNs) which limit the spreading of the virus and accelerate macrophage phagocytosis of viral antigens 28. However, the N protein of SARS-CoV can help the virus escape from the immune responses 29.

Soon, the adaptive immune response joins the fight against the virus. T lymphocytes including CD4^+^ and CD8^+^ T cells play an important role in the defense. CD4^+^ T cells stimulate B cells to produce virus-specific antibodies, and CD8^+^ T cells directly kill virus-infected cells. T helper cells produce proinflammatory cytokines to help the defending cells. However, CoV can inhibit T cell functions by inducing apoptosis of T cells. The humoral immunity including complements such as C3a and C5a and antibodies is also essential in combating the viral infection 30,31. For example, antibodies isolated from a recovered patient neutralized MERS-CoV 32. On the other hand, an overreaction of the immune system generates a large number of free radicals locally that can cause severe damages to the lungs and other organs, and, in the worst scenario, multi-organ failure and even death 33.

## Clinical features

### The incubation periods

The SARS-CoV-2 infection, featured by clustering onset, is more likely to affect elderly people with comorbidities and pregnant women 8. It is common that for people who are exposed to a large number of viruses or whose immune functions are compromised, they have higher chance to be infected than others. The estimated mean incubation period of SARS-CoV-2 is 1-14 days, mostly 3-7 days based on a study of the first 425 cases in Wuhan 36. However, a study on 1,099 cases demonstrates that the incubation period was 3 days on average and ranged from 0 to 24 days 8. A more recent study, as described above, demonstrates that the incubation period was 4.8 (3.0-7.2) days based on the demography of 8,866 cases 37. It is very important for health authorities to adjust the effective quarantine time based on the most accurate incubation period, thus preventing infected but symptomless people from transmitting the virus to others 38. As a common practice, individuals exposed to, or infected by, the virus are usually required to be quarantined for 14 days. Should the quarantine time be extended to 24 days?

### Symptoms

Fever is often the major and initial symptom of COVID-19, which can be accompanied by no symptom or other symptoms such as dry cough, shortness of breath, muscle ache, dizziness, headache, sore throat, rhinorrhea, chest pain, diarrhea, nausea, and vomiting. Some patients experienced dyspnea and/or hypoxemia one week after the onset of the disease 8. In severe cases, patients quickly progressed to develop acute respiratory syndrome, septic shock, metabolic acidosis, and coagulopathy. Patients with fever and/or respiratory symptoms and acute fever, even without pulmonary imaging abnormalities, should be screened for the virus for early diagnosis 39-41.

A demographic study in late December of 2019 showed that the percentages of the symptoms were 98% for fever, 76% for dry cough, 55% for dyspnea, and 3% for diarrhea; 8% of the patients required ventilation support 42. Similar findings were reported in two recent studies of a family cluster and a cluster caused by transmission from an asymptomatic individual 43,44. Comparably, a demographic study in 2012 showed that MERS-CoV patients also had fever (98%), dry cough (47%), and dyspnea (55%) as their main symptoms. However, 80% of them required ventilation support, much more than COVID-19 patients and consistent with the higher lethality of MERS than of COVID-19. Diarrhea (26%) and sore throat (21%) were also observed with MERS patients. In SARS patients, it has been demonstrated that fever (99%-100%), dry cough (29%-75%), dyspnea (40%-42%), diarrhea (20-25%), and sore throat (13-25%) were the major symptoms and ventilation support was required for approximately 14%-20% of the patients 45.

By February 14, the mortality of COVID-19 was 2% when the confirmed cases reached 66,576 globally. Comparably, the mortality of SARS by November 2002 was 10% of 8,096 confirmed cases 46. For MERS, based on a demographic study in June 2012, the mortality was 37% of 2,494 confirmed cases 47. An earlier study reported that the R0 of SARS-CoV-2 was as high as 6.47 with a 95% confidence interval (CI) of 5.71-7.23 48, whereas the R0 of SARS-CoV only ranged from 2 to 4 49. A comparison of SARS-CoV-2 with MERS-CoV and SARA-CoV regarding their symptoms, mortality, and R0 is presented in Table 1. The above figures suggest that SARS-CoV-2 has a higher ability to spread than MERS-CoV and SARS-CoV, but it is less lethal than the latter two 6. Thus, it is much more challenging to control the epidemic of SARS-CoV-2 than those of MERS-CoV and SARS-CoV.

## Diagnosis

### Patient history

Clustered onset often happens in the same family or from the same gathering or vehicle such as a cruise ship. Patients often have a history of travel or residence in Wuhan or other affected areas or contact with infected individuals or patients in the recent two weeks before the onset 50. However, it has been reported that people can carry the virus without symptoms longer than two weeks and cured patients discharged from hospitals can carry the virus again 51, which sends out an alarm to increase the time for quarantine.

### Laboratory results

Patients have normal or reduced number of peripheral white blood cells (especially lymphocytes) at the early stage. For example, lymphopenia with white blood cell count < 4×10^9^/L including lymphocyte count < 1×10^9^/L, and elevated aspartate aminotransferase levels and viremia were found in 1,099 COVID-19 patients 8. The levels of liver and muscle enzymes and myoglobin were increased in the blood of some patients, and C-reactive protein and erythrocyte sedimentation were increased in the blood of most patients 52. In patients with severe cases, the level of D-dimer, a fibrin degradation product present in the blood, was elevated, and lymphocyte count was progressively reduced 34.

### Radiography

Abnormalities in chest radiography are found in most COVID-19 patients and featured by bilateral patchy shadows or ground glass opacity in the lungs. Patients often develop an atypical pneumonia, acute lung injury, and acute respiratory distress syndrome (ARDS) 34. When ARDS happens, uncontrolled inflammation, fluid accumulation, and progressive fibrosis severely compromise the gas exchange. Dysfunction of type-I and type-II pneumocytes decreases the surfactant level and increases surface tension, thus reducing the ability of the lungs to expand and heightening the risk of lung collapse 53,54. Therefore, the worst chest radiographic findings often parallel the most severe extent of the disease 55.

### Pathology

On February 18, 2020, the first pathological analysis of COVID-19 demonstrated the desquamation of pneumocytes, hyaline membrane formation, and interstitial lymphocyte infiltration, and multinucleated syncytial cells in the lungs of a patient who died of the disease, consistent with the pathology of viral infection and ARDS 56 and similar to that of SARS and MERS patients 57,58.

### Nuclear acid assays

The detection of SARS-CoV-2 RNA via reverse-transcriptase polymerase chain reaction (RT-PCR) was used as the major criteria for the diagnosis of COVID-19. However, due to the high false-negative rate, which may accelerate the epidemic, clinical manifestations started to be used for diagnosis (which no longer solely relied on RT-PCR) in China on February 13, 2020. A similar situation also occurred with the diagnosis of SARS 59. Therefore, a combination of disease history, clinical manifestations, laboratory tests, and radiological findings is essential and imperative for making an effective diagnosis. On February 14, 2020, the Feng Zhang group described a protocol of using the CRISPR-based SHERLOCK technique to detect SARS-CoV-2, which detects synthetic SARS-CoV-2 RNA fragments at 20 × 10^-18^ mol/L to 200 × 10^-18^ mol/L (10-100 copies per microliter of input) using a dipstick in less than an hour without requiring elaborate instrumentation 60. Hopefully, the new technique can dramatically enhance the sensitivity and convenience if verified in clinical samples.

## Treatment

Due to the lack of experience with the novel CoV, physicians can mainly provide supportive care to COVID-19 patients, while attempting a variety of therapies that have been used or proposed before for the treatment of other CoVs such as SARS-CoV and MERS-CoV and other viral diseases (Table 2). These therapies include current and potential treatments with antiviral drugs, immunosuppressants, steroids, plasma from recovered patients, Chinese medicine, and psychological support. Even plasma from recovered patients was proposed to be used for treatment 61. Pharmaceutical companies are racing to develop antibodies and vaccines against the virus 62.

### Supportive care

SARS-CoV-2 mainly attacks the lungs in the beginning and probably also attacks, to a lesser degree, other organs that express ACE2, such as the gastrointestinal system and the kidneys. Nevertheless, respiratory dysfunction and failure are the major threat to the patients and the major cause of death. Thus, respiratory support is critical to relieve the symptoms and save lives and includes general oxygen therapy, high-flow oxygen, noninvasive ventilation, and invasive mechanical ventilation depending on the severity of the disease. Patients with severe respiratory symptoms have to be supported by extracorporeal membrane oxygenation (ECMO), a modified cardiopulmonary bypass technique used for the treatment of life-threatening cardiac or respiratory failure. In addition, the maintenance of electrolyte balance, the prevention and treatment of secondary infection and septic shock, and the protection of the functions of the vital organs are also essential for SARS-CoV-2 patients 7.

### Tackling cytokine storms

It has been known that a cytokine storm results from an overreaction of the immune system in SARS and MERS patients 33. Cytokine storm is a form of systemic inflammatory response featured by the release of a series of cytokines including TNFα, IL-1β, IL-2, IL-6, IFNα, IFNβ, IFNγ, and MCP-1. These cytokines induce immune cells to release a vast number of free radicals which are the major cause of ARDS and multiple organ failure 67. Immunosuppression is essential in the treatment of cytokine storms, especially in severe patients.

Corticosteroids and tocilizumab, an anti-IL6 monoclonal antibody, have been used to treat cytokine storm 68. Other immunosuppression treatments for cytokine storm include the modulation of T cell-directed immune response; the blockade of IFN-γ, IL-1, and TNF; JAK inhibition 69; blinatumomab 70; suppressor of cytokine signaling 4 71; and HDAC inhibitors 72.

Steroids, as immunosuppressants, were widely used in the treatment of SARS to reduce the severity of inflammatory damage 65. However, steroids at high dosages were not beneficial to severe lung injury in SARS and COVID-19 patients 59,73. Instead, they may cause severe side effects, especially avascular osteonecrosis, dramatically affecting the prognosis 74. Nevertheless, short courses of corticosteroids at low-to-moderate doses have been recommended to be used prudently for critically ill COVID-19 patients 75.

### Antiviral therapy

At the time of writing, no effective antiviral therapy has been confirmed. However, intravenous administration with remdesivir, a nucleotide analog, has been found to be efficacious in an American patient with COVID-19 64. Remdesivir is a novel antiviral drug developed by Gilead initially for the treatment of diseases caused by Ebola and Marlburg viruses 76. Later, remdesivir also demonstrated possible inhibition of other single stranded RNA viruses including MERS and SARS viruses 77,78. Based on these, Gilead has provided the compound to China to conduct a pair of trials on SARS-CoV-2-infected individuals 79, and the results are highly anticipated.

In addition, baricitinb, interferon-α, lopinavir/ritonavir, and ribavirin have been suggested as potential therapies for patients with acute respiratory symptoms 80,81. Diarrhea, nausea, vomiting, liver damage, and other adverse reactions can occur following combined therapy with lopinavir/ritonavir 80. The interaction of these treatments with other drugs used in the patients should be monitored carefully.

### Plasma from recovered patients and antibody generation

The collection of the blood from patients who recovered from a contagious disease to treat other patients suffering from the same disease or to protect healthy individuals from catching the disease has a long history 82. Indeed, recovered patients often have a relatively high level of antibodies against the pathogen in their blood. Antibodies are an immunoglobulin (Ig) produced by B lymphocytes to fight pathogens and other foreign objects and they recognize unique molecules in the pathogens and neutralize them directly 83. Based on this, plasma was collected from the blood of a group of patients who recovered from COVID-19 and was injected into 10 seriously ill patients. Their symptoms improved within 24 hours, accompanied by reduced inflammation and viral loads and improved oxygen saturation in the blood. However, verification and clarification are necessary to propose the method for large-scale use before specific therapies are not yet developed.

In addition, given the therapeutic effects, some disadvantages associated with the plasma should be considered carefully. For example, antibodies can overstimulate the immune response and cause cytokine release syndrome, which is potentially a life-threatening toxicity 84. The concentration of antibodies in the blood is usually low, and the demand for the plasma is large to treat critically ill patients. It is difficult to develop and produce specific antibodies rapidly enough to fight against a global epidemic 62. Thus, it is more critical and practical to isolate B cells from recovered patients and identify the genetic codes encoding effective antibodies or screen for effective antibodies against essential proteins of the virus. This way, we can readily scale up the production of the antibodies.

### Traditional Chinese medicine (TCM)

TCM has been used to treat a variety of diseases in China for thousands of years. However, its effects largely rely on a combination of multiple components in a formula that varies depending on the diagnosis of a disease based on the theories of TCM. Most of the effective components remain unknown or are vague as it is difficult to extract and verify such components or their optimal combinations. Currently, due to the lack of effective and specific therapy for COVID-19, TCM has become one of the major alternative treatments for patients with light to moderate symptoms or for those who have recovered from severe stages 85. For example, Shu Feng Jie Du capsules and Lian Hua Qing Wen capsules were found to be effective for COVID-19 treatment 86. Top cure rates in the treatment of COVID-19 patients were observed in several provinces in China that used TCM in 87% of their patients, including Gansu (63.7%), Ningxia (50%), and Hunan (50%), whereas Hubei province, which used TCM in only approximately 30% of its COVID-19 patients, had the lowest cure rate (13%) 87. However, this is quite a rough comparison as many other impact factors such as the number and severity of the patients should be included in the evaluation.

On February 18, 2020, Boli Zhang and coworkers published a study to compare western medicine (WM) treatment alone with combined treatment of WM and TCM 88. They found that the times needed for body temperature recovery, symptom disappearance, and hospitalization were remarkably shorter in the WM+TCM group than in the WM only group. Most impressively, the rate for symptomatic worsening (from light to severe) was remarkably lower for the WM+TCM group than for the WM only group (7.4% versus 46.2%) and the mortality was lower in the WM+TCM group than WM only group (8.8% versus 39%). Nevertheless, the efficacy and safety of TCM still await more well-controlled trials at larger scales and in more centers. It would also be intriguing to characterize the mechanism of actions and clarify the effective components of TCM treatments or their combinations if possible.

### Mental health care

Patients with suspected or confirmed COVID-19 mostly experience great fear of the highly contagious and even fatal disease, and quarantined people also experience boredom, loneliness, and anger. Furthermore, symptoms of the infection such as fever, hypoxia, and cough as well as adverse effects of the treatments such as insomnia caused by corticosteroids can lead to more anxiety and mental distress. In the early phase of the SARS outbreak, a range of psychiatric morbidities including persistent depression, anxiety, panic attacks, psychomotor excitement, psychotic symptoms, delirium, and even suicidality were reported 89,90. Mandatory contact tracing and quarantine, as a part of the public health responses to the COVID-19 outbreak, can make people more anxious and guilty about the effects of the contagion, quarantine, and stigma on their families and friends 66.

Thus, mental health care should be provided to COVID-19 patients, suspected individuals, and people in contact with them as well as the general public who are in need. The psychological support should include the establishment of multidisciplinary mental health teams, clear communications with regular and accurate updates about the SARS-CoV-2 outbreak and treatment plans and the use of professional electronic devices and applications to avoid close contact with each other 66.

### Vaccination

Effective vaccines are essential for interrupting the chain of transmission from animal reservoirs and infected humans to susceptible hosts and are often complementary to antiviral treatment in the control of epidemics caused by emerging viruses. Efforts have been made to develop S protein-based vaccines to generate long-term and potent neutralizing antibodies and/or protective immunity against SARS-CoV 81,91. Live-attenuated vaccines have been evaluated in animal models for SARS 92. However, the *in vivo* efficacy of these vaccine candidates in elderly individuals and lethal-challenge models and their protection against zoonotic virus infection have yet to be determined before a clinical study is initiated. This is probably because SARS died down 17 years ago and no new case has been reported since.

In contrast, sporadic cases and clusters of MERS continue to occur in the Middle East and spread to other regions owing to the persistence of zoonotic sources in endemic areas. Vaccination strategies have been developed for MERS by using inactivated virus, DNA plasmids, viral vectors, nanoparticles, virus-like particles and recombinant protein subunits and some have been evaluated in animal models 93. The development of a safe and effective vaccine against SARS-CoV-2 for non-immune individuals is an urgent and critical task for controlling the ongoing epidemic. However, it is challenging to overcome the difficulty because of the long period of time (averaged 18 months) needed for vaccine development and the dynamic variations of CoVs.

## Prognosis

### Prognosis of patients

As a novel disease, COVID-19 has just started to manifest its full clinical course throughout thousands of patients. In most cases, patients can recover gradually without sequelae. However, similar to SARS and MERS, COVID-19 is also associated with high morbidity and mortality in patients with severe cases. Therefore, building a prognosis model for the disease is essential for health-care agencies to prioritize their services, especially in resource-constrained areas. Based on clinical studies reported thus far, the following factors may affect or be associated with the prognosis of COVID-19 patients (Table 3):

- **Age:** Age was the most important factor for the prognosis of SARS 99, which is also true for COVID-19. COVID-19 mainly happened at the age of 30-65 with 47.7% of those patients being over 50 in a study of 8,866 cases as described above 37. Patients who required intensive care were more likely to have underlying comorbidities and complications and were significantly older than those who did not (at the median age of 66 versus 51) 34, suggesting age as a prognostic factor for the outcome of COVID-19 patients.
- **Sex:** SARS-CoV-2 has infected more men than women (0.31/100,000 versus 0.27/100,000), as described above 37.
- **Comorbidities and complications:** Patients with COVID-19 who require intensive care are more likely to suffer from acute cardiac injury and arrhythmia 34. Cardiac events were also the main reason for death in SARS patients 55,65,99. It has been reported that SARS-CoV-2 can also bind to ACE2-positive cholangiocytes, which might lead to liver dysfunctions in COVID-19 patients 100. It is worth noting that age and underlying disease are strongly correlated and might interfere with each other 55.
- **Abnormal laboratory findings**: The C-reactive protein (CRP) level in blood reflects the severity of inflammation or tissue injury and has been proposed to be a potential prognostic factor for disease, response to therapy, and ultimate recovery 101. The correlation of CRP level to the severity and prognosis of COVID-19 has also been proposed 101. In addition, elevated lactate dehydrogenase (LDH), aspartate aminotransferase (AST), alanine aminotransferase (ALT), and creatine kinase (CK) may also help predict the outcome. These enzymes are expressed extensively in multiple organs, especially in the heart and liver, and are released during tissue damage 102,103. Thus, they are traditional markers for heart or liver dysfunctions.
- **Major clinical symptoms:** Chest radiography and temporal progression of clinical symptoms should be considered together with the other issues for the prediction of outcomes and complications of COVID-19.
- **Use of steroids:** As described above, steroids are immunosuppressant commonly used as an adjunctive therapy for infectious diseases to reduce the severity of inflammatory damage 104. Since a high dosage of corticosteroids was widely used in severe SARS patients, many survivors suffered from avascular osteonecrosis with life-long disability and poor life quality 105. Thus, if needed, steroids should be used at low dosage and for a short time in COVID-19 patients.
- **Mental stress:** As described above, during the COVID-19 outbreak many patients have suffered from extraordinary stress as they often endured long periods of quarantine and extreme uncertainty and witnessed the death of close family members and fellow patients. It is imperative to provide psychological counseling and long-term support to help these patients recover from the stress and return to normal life 66.

### Prognosis of the epidemic

According to demographic studies so far, COVID-19 seems to have different epidemiological features from SARS. In addition to replicating in the lower respiratory tract, SARS-CoV-2 can efficiently replicate in the upper respiratory tract and causes mild or no symptoms in the early phase of infection, similar to other CoVs that cause common colds 106. Therefore, infected patients at the early phase or incubation period can produce a large amount of virus during daily activities, causing great difficulty for the control of the epidemic. However, the transmission of SARS-CoV was considered to occur when the patients are severely ill, while most transmission did not happen at the early phase 107. Thus, the current outbreak of COVID-19 is much more severe and difficult to control than the outbreak of SARS.

Great efforts are currently underway in China including the lockdown of Wuhan and surrounding cities and continuous quarantine of almost the entire population in hopes of interrupting the transmission of SARS-CoV-2. Although these actions have been dramatically damaging the economy and other sectors of the country, the number of new patients is declining, indicating the slowdown of the epidemic. The most optimistic estimate is that the outbreak will end by March and the downswing phase will last for 3-4 months 108. However, some other experts are not that optimistic. Paul Hunter, *et al.*, estimated that COVID-19, which seems substantially more infectious than SARS, will not end in 2020 109. Ira Longini, *et al.*, established a model to predict the outcome of the epidemic and suggested that SARS-CoV-2 could infect two-thirds of the global population 110.

**Figure 5:**
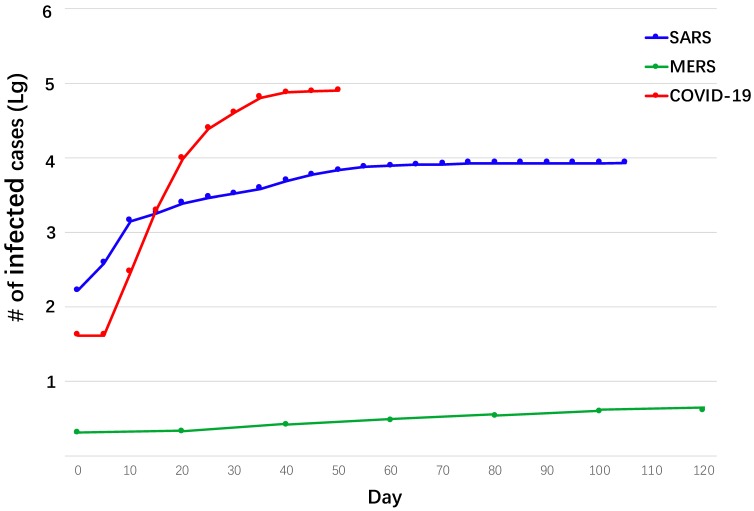
Comparison of epidemic of COVID-19, SARS, and MERS.

A Canadian group reported that SARS-CoV-2 was detected in both mid-turbinate and throat swabs of patients who recovered and left the hospital 2 weeks earlier 111, which indicates that the newly identified virus could become a cyclical episode similar to influenza. However, promising signs have occurred in China based on the declining number of new cases, indicating the current strategies might have been working. Ebola was originally predicted to cause up to a million cases with half a million deaths. However, via strict quarantine and isolation, the disease has eventually been put under control 112,113. It is possible, similar to SARS-CoV, that SARS-CoV-2 might become weaker in infectivity and eventually die down or become a less pathogenic virus co-existent with humans. A comparison of the epidemic of COVID-19 with that of SARS and MERS is provided below (Fig. 5).

## Prevention

SARS-CoV-2 is highly transmittable through coughing or sneezing, and possibly also through direct contact with materials contaminated by the virus 12. The virus was also found in feces, which raises a new possibility of feces-to-mouth transmission 114. A recent study on 138 cases reported that 41% of the cases were possibly caused by nosocomial infections, including 17 patients with other prior diseases and 40 health-care providers 115. Thus, great precaution should be used to protect humans, especially health-care providers, social workers, family members, colleagues, and even bystanders in contact with patients or infected people.

### Personal role

The first line of defense that could be used to lower the risk of infection is through wearing face masks; both the use of surgical masks and N95 respirator masks (series # 1860s) helps control the spread of viruses 116. Surgical face masks prevent liquid droplets from a potentially infected individual from traveling through the air or sticking onto surfaces of materials, where they could be passed on to others 117. However, only N95 (series # 1860s) masks can protect against the inhalation of virions as small as 10 to 80 nm, with only 5% of the virions being able to penetrate completely; SARS-CoV-2 is similar to SARS-CoV in size and both are approximately 85 nm 117. Since particles can penetrate even five surgical masks stacked together, health-care providers in direct contact with patients must wear N95 (series # 1860s) masks but not surgical masks 118.

In addition to masks, health-care providers should wear fitted isolation gowns in order to further reduce contact with viruses. Viruses can also infect an individual through the eyes. On January 22, 2020, a doctor was infected with SARS-CoV-2 although he wore an N95 mask; the virus might have entered his body through his inflammatory eyes 119. Thus, health-care providers should also wear transparent face shields or goggles while working with patients.

For the general public in affected or potentially affected areas, it is highly suggested that everybody wash their hands with disinfectant soaps more often than usual, try to stay indoors for self-quarantine and limit contact with potentially infected individuals. Three feet is considered an appropriate distance for people to stay away from a patient 120. These actions are effective methods to lower the risk of infection as well as prevent the spread of the virus.

### Governmental role

Although SARS-CoV-2 came as a new virus to the human world, its high homology to SARS-CoV as reported on 7 January 2020 3 should have caused high alert to China based on her deep memory of the SARS outbreak in 2003. However, not until 19 January 2020 did the director of the Center of Disease Control of Wuhan comfort the citizens by saying that the novel virus has low contagiousness and limited reproductivity from human to human and that it is not a problem to prevent and contain the disease. This message remarkably relaxed the alarm of the public, especially when the entire country was preparing for the Spring Festival, and the critical time was missed to contain the disease at its minimal scale in Wuhan.

The disease control agencies in China may take this hard lesson and make critical improvements in the future. For example, these agencies should be (1) more careful when making public announcements as every word counts to citizens and can change their attitude and decisions; (2) more sensitive and reactive to unusual information from clinics rather than waiting for formal reports from doctors or officials; (3) more restrictive to contain a potential epidemic at its early stage rather than attempting to comfort the public; and (4) more often to issue targeted and effective drills to increase the public's awareness about epidemic diseases and to test and improve the response system of the society periodically.

## Concluding remarks

The outbreak of COVID-19 caused by the novel virus SARS-CoV-2 started in the end of December 2019. In less than two months, it has spread all over China and near 50 other countries globally at the time of this writing. Since the virus is very similar to SARS-CoV and the symptoms are also similar between COVID-19 and SARS, the outbreak of COVID-19 has created a sense of SARS recurring. However, there are some remarkable differences between COVID-19 and SARS, which are essential for containing the epidemic and treating the patients.

- COVID-19 affects more elderly individuals than youth and more men than women, and the severity and death rate are also higher in elderly individual than in youth.
- SARS has higher mortality than COVID-19 (10.91% versus 1.44%).
- COVID-19 patients transmit the virus even when they are symptomless whereas SARS patients do so usually when they are severely ill, which causes much greater difficulty to contain the spread of COVID-19 than SARS. This partially explains why SARS-CoV-2 spread much faster and broader than SARS-CoV.
- The regular RNA assay for SARS-CoV-2 can be negative in some COVID-19 patients. On the other hand, cured patients can be positive for the virus again. These findings dramatically increase the risk of virus spreading.

Given such rapid progress in research on COVID-19, several critical issues remain to be solved, as follows:

- Where did SARS-CoV-2 come from? Although 96% genetic homolog was found between SARS-CoV-2 and two bat SARS-like CoVs, we still cannot conclude that SARS-CoV-2 is from bats.
- What animal was the intermediate species to transmit the virus from the original host, say bats, to humans? Without knowing answers to #1 and 2, we cannot efficiently cut the transmission, and the outbreak can relapse at any time.
- Although molecular modeling and biochemical assays have demonstrated that SARS-CoV-2 binds to ACE2, how exactly does the virus enter the airway cells and cause subsequent pathological changes? Does the virus also bind ACE2-expressing cells in other organs 121? Without clear answers to these questions, we cannot achieve fast and accurate diagnosis and effective treatment.
- How long will the epidemic last? How is the virus genetically evolving during transmission among humans? Will it become a pandemic worldwide, die down like SARS or relapse periodically like the flu?

It is essential but may take some time to search for answers to the above and many other questions. However, with whatever expense it may demand, we have no other choice but to stop the epidemic as soon as possible and bring our life back to normal.

## Figures and Tables

**Table 1:** Comparison of SARS-CoV-2 with SARS-CoV and MERS-CoV

Comparison items	SARS-CoV-2	MERS-CoV	SARS-CoV	Reference
**Characteristics**				6,37
Date extracted from	Dec. 2020 - Feb. 2020	Sept. 2012	Dec. 2003	
Place of origin	Wuhan, China	Jeddah, Saudi Arabia	Guangdong China	
Age range	56 (22-92)	56 (14-94)	39.9 (1-91)	
Male/Female sex ratio	1:3:1	3.3:1	1.1.25	
Mortality rate	2%	34%	9.6%	
Confirmed cases (Global)	24554	2494	8096	
R_0_	4.7 - 6.6	0.45 - 0.91	0.86 - 1.88	37,42
Incubation period (day)	7 - 14	5.0 - 6.9	4.4 - 6.9	
**Symptoms**				6,34,37
Fever	258 (93%)	98%	99-100%	
Dry cough	194 (70%)	47%	29-75%	
Dyspnea	96 (35%)	72%	40-42%	
Diarrhea	17 (6%)	26%	20-25%	
Sore throat	10 (4%)	21%	13-25%	

**Table 2:** Treatments of COVID-19

Treatment	Efficacy	References
General oxygen therapy, high-flow oxygen/noninvasive ventilation, and invasive mechanical ventilation, conservation fluid management, management of septic shock, infection control	Respiratory supports	7
Antiviral therapy	Reduce the viral load	63
Remdesivir	-	64
Steroids	Reduce the severity of inflammatory damage	65
Psychological supports	Relieve stress and improve mental health	66

**Table 3:** Prognostic factors for COVID-19 in comparison with SARS and MERS

Disease	Laboratory results	Chest radiography	Others	Reference
COVID-19	CRP AST ALT CK LDH D-dimer Lymphopenia	Chest radiography	Age Gender Pregnancy Viral load Underlying disease	
SARS	CRP AST ALT CK LDH D-dimer Low platelet Plasma electrolyte	Chest radiography	Age Gender Pregnancy Underlying diseases	55, 59, 65, 94
MERS	Neutrophilia Lymphopenia Serum creatinine LDH	The diffuse or brochopneumonia	Age, Chronic kidney disease, Hypertension, Viral load	95-98
